# A paclitaxel and microRNA-124 coloaded stepped cleavable nanosystem against triple negative breast cancer

**DOI:** 10.1186/s12951-021-00800-z

**Published:** 2021-02-25

**Authors:** Chuanrong Chen, Ming Shen, Hongze Liao, Qianqian Guo, Hao Fu, Jian Yu, Yourong Duan

**Affiliations:** 1 State Key Laboratory of Oncogenes and Related Genes, Shanghai Cancer Institute, Renji Hospital, School of Medicine, Shanghai Jiao Tong University, Shanghai, 200032 China; 2 NHC Key Laboratory of Reproduction Regulation, (Shanghai Institute of Planned Parenthood Research), Fudan University, and Shanghai Engineer and Technology Research Center of Reproductive Health Drug and Devices, Shanghai, 200032 China; 3 Research Center for Marine Drugs, State Key Laboratory of Oncogene and Related Genes, Department of Pharmacy, Ren Ji Hospital, School of Medicine, Shanghai Jiao Tong University, Shanghai, 200127 China; 4 State Key Laboratory of Oncogenes and Related Genes, Renji Hospital, School of Biomedical Engineering, Shanghai Jiao Tong University, Shanghai, 200127 China

**Keywords:** Stepped cleavable nanoparticles, Paclitaxel, MicroRNA-124, Synergistic antitumour effect, Triple negative breast cancer

## Abstract

**Background:**

Triple negative breast cancer (TNBC) is one of the most biologically aggressive breast cancers and lacks effective treatment options, resulting in a poor prognosis. Therefore, studies aiming to explore new therapeutic strategies for advanced TNBC are urgently needed. According to recent studies, microRNA-124 (miR124) not only inhibits tumour growth but also increases the sensitivity of TNBC to paclitaxel (PTX), suggesting that a platform combining PTX and miR124 may be an advanced solution for TNBC.

**Results:**

Herein, we constructed a stepped cleavable calcium phosphate composite lipid nanosystem (CaP/LNS) to codeliver PTX and miR124 (PTX/miR124-NP). PTX/miR124-NP exhibited superior tumor microenvironment responsive ability, in which the surface PEG layer was shed in the mildly acidic environment of tumor tissues and exposed oligomeric hyaluronic acid (o-HA) facilitated the cellular uptake of CaP/LNS by targeting the CD44 receptor on the surface of tumor cells. Inside tumour cells, o-HA detached from CaP/LNS due to the reduction of disulfide bonds by glutathione (GSH) and inhibited tumour metastasis. Then, PTX and miR124 were sequentially released from CaP/LNS and exerted synergistic antitumour effects by reversing the Epithelial-Mesenchymal Transition (EMT) process in MDA-MB-231 cells. Moreover, PTX/miR124-NP showed significant antitumour efficiency and excellent safety in mice bearing MDA-MB-231 tumours.

**Conclusion:**

Based on these results, the codelivery of PTX and miR124 by the CaP/LNS nanosystem might be a promising therapeutic strategy for TNBC.
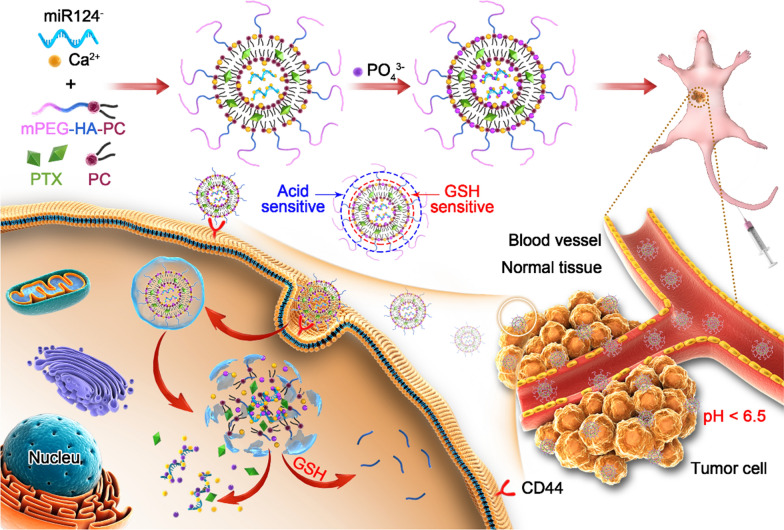

## Background

Breast cancer is a major disease that threatens human life [1]. TNBC is a type of breast cancer that does not express the estrogen receptor (ER), progesterone receptor (PR) or human epidermal growth factor receptor 2 (HER-2), and accounts for 15–20% of all breast cancers [1, 2]. It always shows strong invasiveness and results in a poor prognosis, because it presents an invasive course in the clinic with a high recurrence rate within 1–3 years and results in high distant metastasis and mortality rates [3–5].

Due to its special molecular biological characteristics (ER, PR, and HER-2 negative), endocrine therapy and anti-HER-2 targeted therapy are not suitable for TNBC [6]. Chemotherapy is still the main treatment strategy for TNBC. Paclitaxel is a first-line chemotherapy drug for TNBC, but its efficacy is unsatisfactory when used alone or in combination with other chemotherapy drugs [7]. Therefore, chemotherapy alone is insufficient for TNBC. MicroRNAs are a group of single-stranded, noncoding RNAs of approximately 22 nucleotides in length that are widely found in eukaryotes. They regulate the expression of target mRNAs mainly by completely or partially complementing the 3′ untranslated region of the target mRNA, resulting in degradation or posttranscriptional translational inhibition of the target mRNA [8]. Notably, miR124, a multifunctional antitumour miRNA, is downregulated in TNBC, and decreased expression of miR124 is an independent unfavourable prognostic factor [9, 10]. Overexpression of miR124 effectively inhibits TNBC growth and metastasis [11–13]. Moreover, miR124 promotes sensitivity to PTX in breast cancer cells [14, 15]. Therefore, the combination of PTX and miR124 may be a superior strategy for the treatment of TNBC.

Shortcomings, such as instability, immunogenicity and difficult internalization by cells, limit the applications of miRNAs in vivo [16, 17]. In addition, free PTX lacks the ability to target tumours in vivo, which leads to decreased efficacy and severe side effects [18, 19]. A tumour-targeting nanodelivery system is a good choice to overcome these shortcomings [20, 21]. Liposomes are commonly used nanosystems with a large entrapment capacity suitable for both water-soluble and fat-soluble drugs with good biocompatibility [22, 23]. Recent advances in calcium phosphate (CaP) composite liposomes overcome two disadvantages of liposomes: drug leakage and reticuloendothelial system (RES) clearance [24]. CaP is a biocompatible material that has been widely used in biomedical engineering, such as bone cement [24, 25]. CaP has also been shown to improve the stability of liposomes and increase the loading efficiency of RNA, prolonging circulation in vivo [26–28]. Therefore, it is a promising approach to realize combinational therapy by preparing PTX and miR124 coloaded CaP liposomes.、

Surface hydrophilic modifications, such as PEGylation, can reduce phagocytosis of the NPs by the RES system, enhance the penetration and retention (EPR) effect and improve biocompatibility [29]. However, the "PEG dilemma" indicates that surface PEGylation strongly inhibits cellular uptake and lysosomal escape, leading to less efficiency of modified NPs [30, 31]. The characteristics of the tumour microenvironment (TME) are significantly different from the normal tissue, such as hypoxia, low pH, and high intracellular GSH levels. [32, 33]. Some drug delivery systems were designed in response to the particular TME and enhanced the antitumour effect [34, 35]. Hence, the stepwise separation of NPs based on the unique TME may solve the "PEG dilemma".

Based on the aforementioned studies, we designed a calcium phosphorus composite lipid nanosystem (CaP/LNS) for PTX and miR124 encapsulation. As designed, the CaP/LNS will be enriched at the tumour site by the long circulation and EPR effect of mPEG after intravenous administration. Then, the mPEG ligand will be removed after the hydrazone bond is hydrolysed in the acidic microenvironment of the tumour tissue to expose o-HA, and the exposed o-HA targets the CD44 receptor on the surface of tumour cells, which facilitates the cellular uptake of CaP/LNS. The disulfide bond is cleaved intracellularly in a high glutathione microenvironment. Thus, o-HA is released into the cytoplasm and antagonizes endogenous HA, which reduces tumour invasiveness. PTX and miR124 are released from CaP/LNS to exert synergistic antitumour effects. This delivery system was prepared in the present study, and its antitumour effects were evaluated in vitro and in vivo.

## Results

### Synthesis of mPEG-HA-PC

The synthetic route of mPEG-HA-PC is shown in Additional file 1: Fig. S1. The function of HA on tumour cells is related to its molecular weight, and o-HA with a molecular weight of approximately 2000 Da increases the adhesion of tumour cells and inhibits tumour cell invasion and metastasis [36]. Therefore, o-HA was used to synthesize mPEG-HA-PC. A hydrazone bond was used to connect mPEG and o-HA, followed by the linkage of o-HA and PC through a disulfide bond. mPEG-HA-PC together with phosphatidylcholine (PC), cholesterol and CaP was prepared into a calcium phosphate composite lipid nanosystem (CaP/LNS). The characteristic shift spectra of mPEG and o-HA were observed in the ^1^H NMR spectra of mPEG-HA (Additional file 1: Fig. S2). The δ 3.291 signal belongs to the β-hydrogen of the hydrazone bond. As shown in Additional file 1: Fig. S3, the absorptions of mPEG and HA were found in the FTIR spectrum of mPEG-HA. The results suggested that mPEG and HA were linked by a hydrazone bond. As shown in Additional file 1: Fig. S4, the stretching peak of an ester C = O (1720 cm^−1^) indicated that mPEG-HA was conjugated with PC. We chose mPEG and HA ratios of 1:1, 2:1, and 4:1 for the nanosystem. As the proportion of mPEG increased, the active hydrogen absorption peaks in the FTIR spectra gradually decreased, while the characteristic C-O stretching peak of mPEG increased.

### Optimized mPEG-HA-PC showed the best pH and GSH sensitivity and invasion inhibition, along with good cell compatibility

**Fig. 1:**
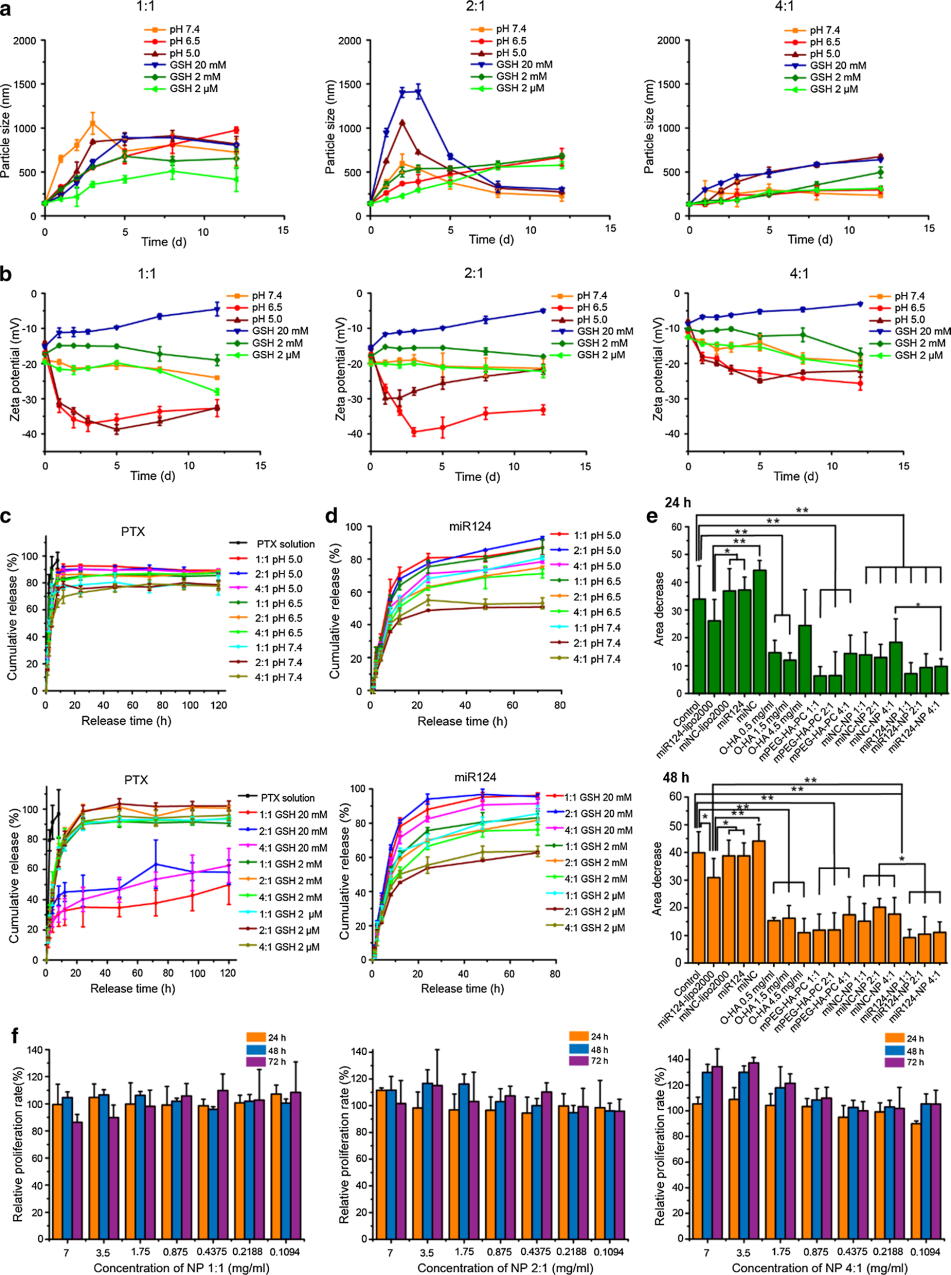
Environmental sensitivity, inhibition of cell migration and biocompatibility of the NPs composed of different PEG-HA ratios. **a** Environmentally sensitive changes in particle size associated with mPEG and the o-HA block ratio. **b** Environmentally sensitive changes in zeta potential associated with mPEG and the o-HA block ratio. **c** Environmentally sensitive release profile of PTX from the NPs (upper, different pH environments; lower, different GSH environments). **d** Environmentally sensitive release profile of miR124 from the NPs (upper, different pH environments; lower, different GSH environments). **e** Effects of different treatments for 24 h (upper) and 48 h (lower) on the migration of MDA-MB-231 cells. (f) Biocompatibility of the NPs composed of the 3 materials in LO2 cells. *p < 0.05, **p < 0.01

The hydrophilic PEG and o-HA layers affect not only their functions but also their sensitive environmental responsiveness. Therefore, we synthesized three mPEG-HA-PC materials with different block ratios (mPEG-HA ratios of 1:1, 2:1, and 4:1) and prepared NPs. The pH and GSH sensitivities, antitumour functions and cell compatibility are shown in Fig. 1.

The particle size, zeta potential and drug release were investigated to optimize the environmental sensitivity of mPEG-HA-PC (Fig. 1a–d). In acidic environments (pH 5.0), the mPEG block should quickly fall apart due to hydrazone bond cleavage, resulting in a decrease in surface hydrophilicity. Particle agglomeration and a zeta potential change followed. Then, due to the dissociation of CaP in an acidic environment, the NPs break down, leading to a decrease in the particle size. When the concentration of GSH was high (20 mM), such as in the tumour cell environment, the disulfide bond between the HA block and PC should be destroyed. The zeta potential would increase because electronegative o-HA would leave. The NPs first agglomerated and then gradually dissociated in the acidic environment of the tumour cells. The normal physiological environment causes almost none of these changes. The results showed that the NPs prepared with a mPEG-HA ratio of 2:1 matched our expectations best.

The release profile of miR124 (cy5-miRNC instead of miR124) from the NPs was faster in acidic and high GSH medium than in a simulated normal body environment. The release of PTX in acidic medium was faster than in a normal body environment. However, a similar change in medium with different GSH concentrations was not observed compared with the change observed for miR124. We speculated that this situation is related to the solubility properties of the two components and their entrapment sites within the NPs. PTX is mainly embedded in the lipid layer, while miR124 is located in the inner water phase. miR124 was released after the NPs were damaged, but the release of PTX was consistent with lipid degradation.

The NPs composed of materials with different PEG-HA ratios showed varying degrees of environmental sensitivity. The 2:1 NPs (prepared with a mPEG-HA ratio of 2:1) displayed the most notable change in particle size and zeta potential in acidic and high GSH media. The miR124 release characteristics of this formulation also showed the most notable environmental responsiveness.

The wound healing assay was performed to optimize the effect of mPEG-HA-PC on inhibiting tumour migration, and Lipo2000 was used as a positive control (Fig. 1e and Additional file 1: Fig. S5). The o-HA block in the material functions as a group that targets tumour cells and inhibits tumour cell migration and invasion. The mPEG block has the effects of increasing surface hydrophilicity and prolonging circulation time in vivo, but it also influences the uptake of NPs by tumour cells. Therefore, it is necessary to choose the best mPEG-HA ratio for application. The inhibitory effect on migration, which was optimal at 1.5 mg/ml, was not linear with the o-HA concentration. The inhibitory effects of either the materials or the NPs composed of ratios of 1:1 and 2:1 were slightly better than NPs with the 1:4 ratio, but no significant differences were observed. Biocompatibility is also a vital parameter to evaluate when selecting a material. The NPs prepared from the three materials did not exhibit significant toxicity to LO2 cells (Fig. 1f; maximum material concentration of 7 mg/ml, relative proliferation rate > 80%).

Based on the results described above, mPEG-HA-PC 2:1 was chosen to prepare the drug delivery system for subsequent studies.

### The optimized synergistic ratio of PTX and miR124

As shown in Additional file 1: Fig. S6, a synergistic effect was observed for most of the selected concentration ranges (CDI < 0.7). The optimized synergistic effect (CDI at 48 h = 0.1929, CDI at 72 h = 0.0964) was observed for 200 pmol/ml miR124 and 0.5 μg/ml PTX. However, since RNA is expensive and difficult to entrap, the concentration of miR124 was selected to be 100 pmol/ml on the premise that the CDIs of 100 pmol/ml:0.1 μg/ml and 100 pmol/ml:0.5 μg/ml were also relatively low. Due to the greater cytotoxicity of 100 pmol/ml:0.5 μg/ml, we chose 100 pmol/ml:0.5 μg/ml as the best ratio.

### Characteristics of the optimized NPs

**Fig. 2:**
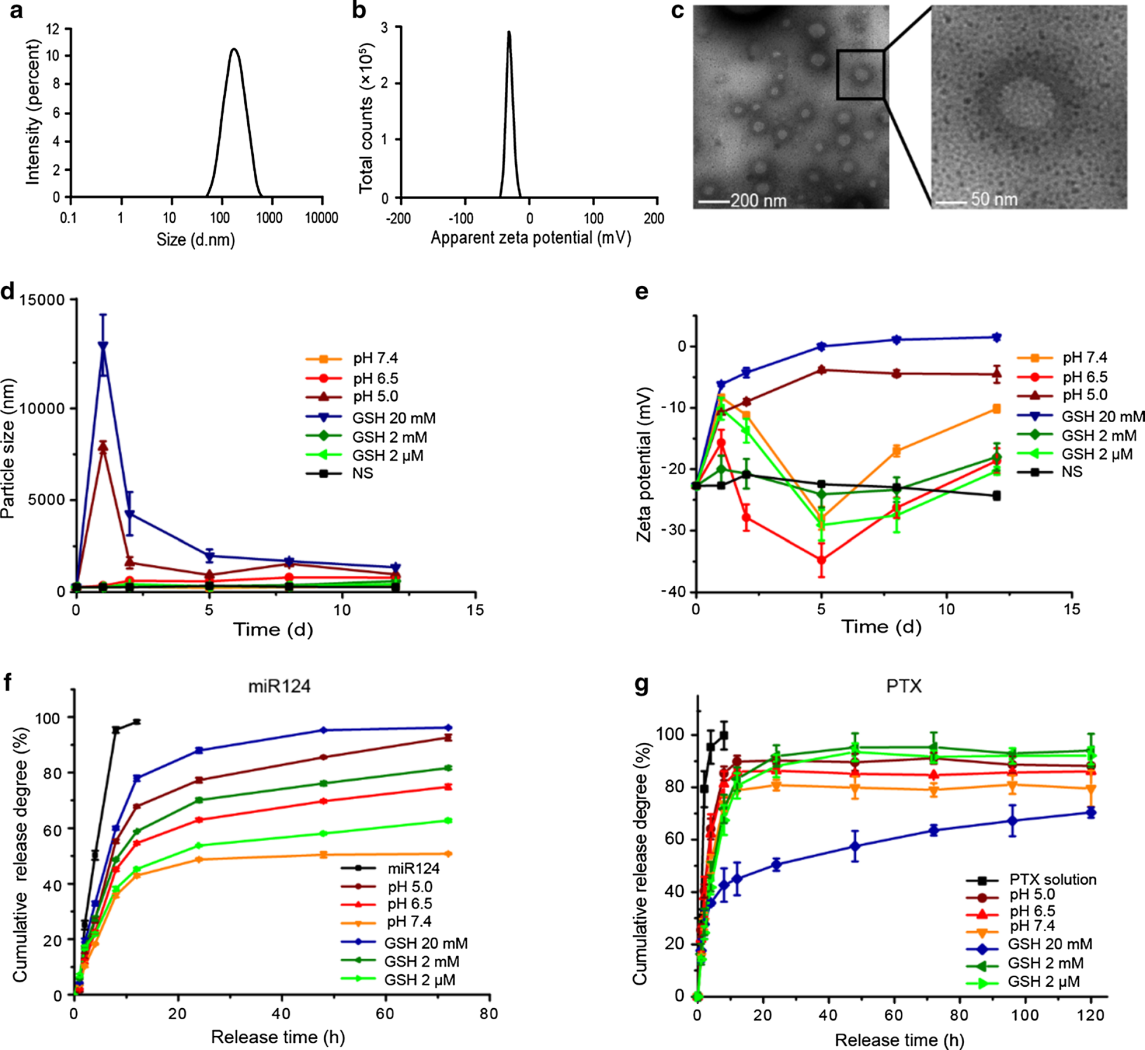
Characterization of PTX and miR124 coloaded CaP/LNS NPs**. a** The hydrodynamic size of the NPs was 187.4 ± 5.6 nm and the PDI was 0.138. **b** Zeta potential distribution of the NPs. The average potential of the NPs was -27.0 ± 3.5 mV. **c** TEM image of the NPs. **d, e** Changes in the hydrodynamic size (d) and zeta potential (e) of the NPs over 12 days in media with different pH values (7.4, 6.5, and 5.0) or GSH (20 mM, 2 mM, and 2 μM) concentrations. **f, g** Release profiles of miR124 (f) and PTX (g) from NPs in environments with different pH values or GSH concentrations

After mPEG-HA-PC 2:1 was chosen for subsequent studies, the method for preparing the NPs was optimized with orthogonal experiments. The characteristics of the optimized NPs are shown in Fig. 2. The particle size was 187.4 ± 5.6 nm with a PDI of 0.138 (Fig. 2a). The zeta potential was -27.0 ± 3.5 mV (Fig. 2b). The encapsulation efficiencies (EEs) of PTX and the miR124 were 92.13% and 89.13%, respectively. The NPs were spherical with a double-layer membrane structure outside. A dark hydration layer around the NPs was visible (Fig. 2c). The size and zeta potential of the optimized NPs maintained good stability in normal saline (NS) for at least 12 days. As expected, at low pH (pH 5.0) and high concentrations of GSH (20 mM), the NPs first aggregated and then dissociated. However, in weakly acidic (pH 6.5) buffer, the cleavage of hydrazone was not as fast as in pH 5.0 buffer. o-HA was exposed slowly, showing a gradual increase in electronegativity. The electronegativity was sufficient to maintain a relatively stable particle size, which helped the NPs maintain mobility in the tumour tissue (Fig. 2d, e).

The release of miR124 showed significant environmental responsiveness and was accelerated under acidic conditions and in the presence of a high GSH concentration (Fig. 2f). The release of PTX showed no obvious correlation with pH and the GSH concentration. The PTX release was slower at pH 7.4 and in the presence of high GSH concentrations (Fig. 2g). This phenomenon may contribute to the antitumour effects in vivo: a much slower release in normal tissues was observed to reduce toxicity, target aggregation at the tumour site, and sustain release in the tumour cells for a long-lasting therapeutic effect.

### Cellular uptake of NPs

**Fig. 3:**
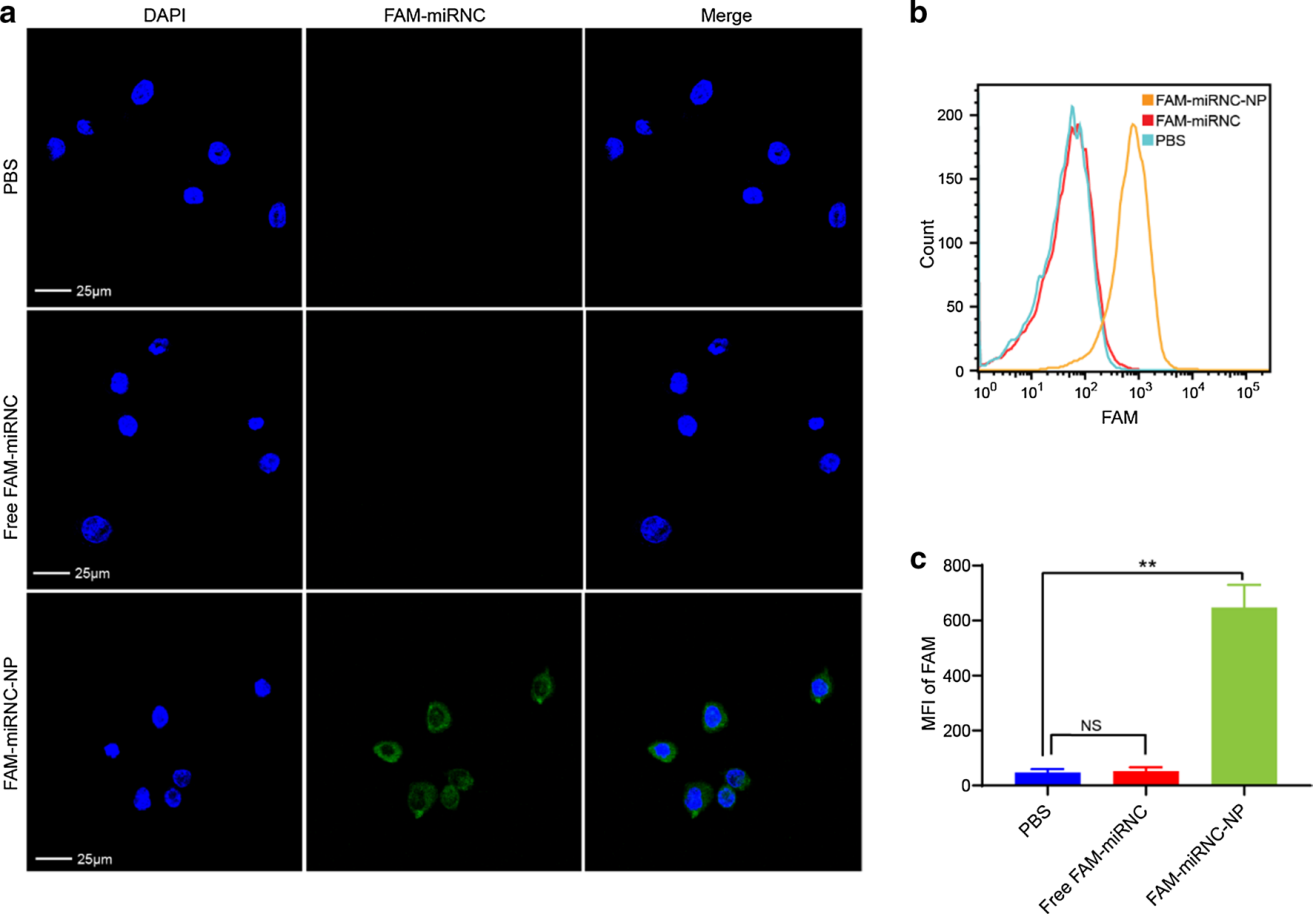
CaP/LNS NPs efficiently transfected miRNA into MDA-MB-231 cells. **a** Representative CLSM images of MDA-MB-231 cells after a 5 h incubation with PBS, free FAM-miRNC or FAM-miRNC-NP. **b** Flow cytometric analysis of FAM-miRNC positive MDA-MB-231 breast cancer cells treated with PBS, free FAM-miRNC or FAM-miRNC-NP. **c** MFI of FAM in MDA-MB-231 cells treated as described above. NS indicates no statistical significance, **p < 0.01

Since RNA is negatively charged and has a high molecular weight, it is not easily internalized by cells [37–39]. Herein, we attempted to codeliver miR124 and PTX into MDA-MB-231 cells using CaP/LNS. Confocal laser scanning microscopy (CLSM) and flow cytometry were used to evaluate the efficiency of NPs uptake by tumour cells. The miRNA negative control was labelled with FAM (FAM-miRNC) as a fluorescent probe. MDA-MB-231 cells were cocultured with PBS, free FAM-miRNC or FAM-miRNC-NP for 5 h. As shown in Fig. 3a, the NPs treated cells exhibited strong fluorescence intensity from FAM-miRNC (green fluorescence), while free FAM-miRNC was rarely taken up by the cells. In addition, according to the flow cytometry data, the mean fluorescence intensity (MFI) of FAM in the NPs group was significantly higher than that in the free and PBS groups, and the MFI in the PBS group and free FAM-miRNC group showed no significant differences (Fig. 3b, c), consistent with the CLSM results. These findings indicated that CaP/LNS NPs efficiently transfected the miRNA into MDA-MB-231 cells.

### In vitro* antitumour effects*

**Fig. 4:**
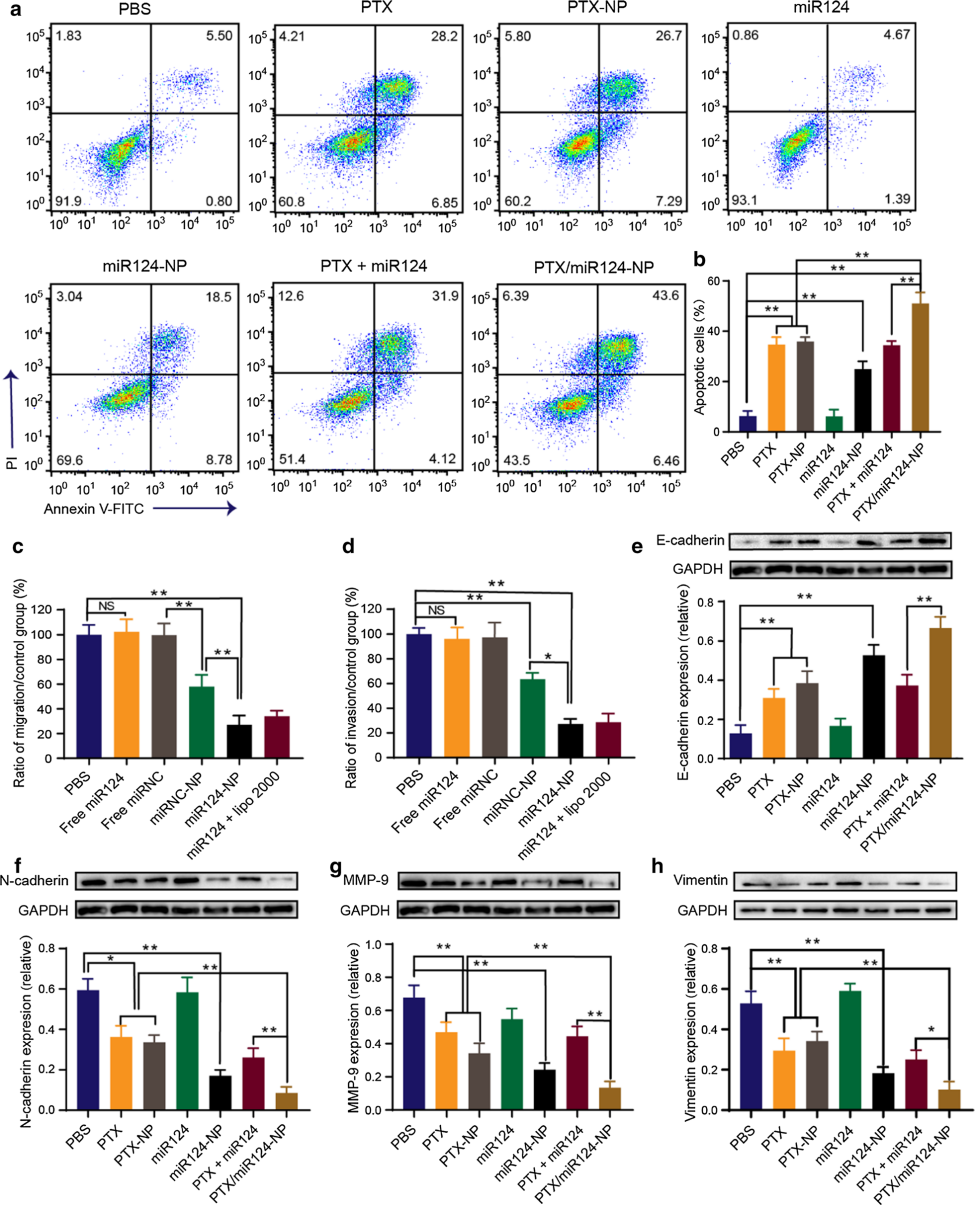
Proapoptotic and antimetastatic effects of PTX/miR124-NP on MDA-MB-231 cells. **a** Flow cytometry analysis of apoptotic MDA-MB-231 cells treated with PBS, PTX, PTX-NP, miR124, miR124-NP, PTX plus miR124 or PTX/miR124-NP for 48 h. **b** Statistical analysis of apoptotic cells (annexin V-FITC positive). **c, d** Cell migration and invasion were detected using Transwell assays. Cells were treated with PBS, free miR124, free miRNC, miRNC-NP, miR124-NP or miR124 + Lipo2000 for 48 h. The ratios of migrating (**c**) and invading cells (**d**) are shown. **e–h** Western blot results for the levels of the EMT markers E-cadherin (**e**), N-cadherin (**f**), MMP-9 (**g**) and Vimentin (**h**) in MDA-MB-231 cells exposed to different treatments for 48 h. NS indicates no statistical significance, *P < 0.05, **P < 0.01

Flow cytometry was performed to evaluate the apoptosis of MDA-MB-231 cells after different treatments. Free miR124 did not induce apoptosis, but the proportion of apoptotic cells increased after transfection with NPs (Fig. 4a, b). No significant difference was observed in the proportion of apoptotic cells between the free PTX and PTX-NP groups, which was attributed to the property of PTX as a low molecular weight lipophilic drug that simply diffuses into cells in vitro. However, the antitumour effects of PTX can be improved in vivo with the assistance of the targeting ability from the NPs. Furthermore, PTX/miR124-NP showed stronger induction of apoptosis than the individual drugs.

Transwell assays were performed to evaluate the effects of miR124 loaded NPs on the migration and invasion of MDA-MB-231 cells. Because the strong cytotoxicity of PTX would reduce cellular activity, PTX was not incorporated into the NPs for the Transwell assay. The number of MDA-MB-231 cells passing through the membrane significantly decreased after treatment with miR124-NP for 48 h. The migration and invasion rates of the miR124-NP group were 27.4% and 11.9%, respectively, and these indexes were the lowest among all groups (Fig. 4c, d). In addition, miRNC-NP exerted an inhibitory effect on cell migration and invasion, suggesting the antimetastatic effect of o-HA.

The EMT has been deemed one of the key mechanisms of tumour progression, invasiveness, metastasis, and chemoresistance [40]. During the EMT, E-cadherin, the key determiner of the epithelial phenotype, is downregulated, while mesenchymal markers (*e.g.,* N-cadherin and Vimentin) are upregulated [41, 42]. Matrix metalloproteinase-9 (MMP-9), one of the most representative matrix metalloproteinases, promotes tumour invasion and metastasis by degrading the extracellular matrix [43, 44]. N-cadherin leads to the overexpression of MMP-9, thereby increasing the cell invasion potential [45]. Emerging evidence indicates that MMP-9 is involved in the development of the EMT and is also an important marker of the EMT process [46, 47]. In the present study, Western blotting results demonstrated that the expression of the epithelial phenotypic protein E-cadherin increased and the expression of mesenchymal phenotypic proteins N-cadherin, MMP-9 and Vimentin decreased in each treatment group except for the free miR124 group (Fig. 4e–h). Changes in the expression of these proteins confirmed reversion of the EMT process. More importantly, the inhibition of EMT in the PTX/miR124 -NP group was stronger than that in the other treatment groups. The results indicated that codelivery of PTX and miR124 enhanced reversal of the EMT process, thus improving efficiency against TNBC.

### *Antitumour efficacy of PTX/miR124-NP *in vivo

**Fig. 5:**
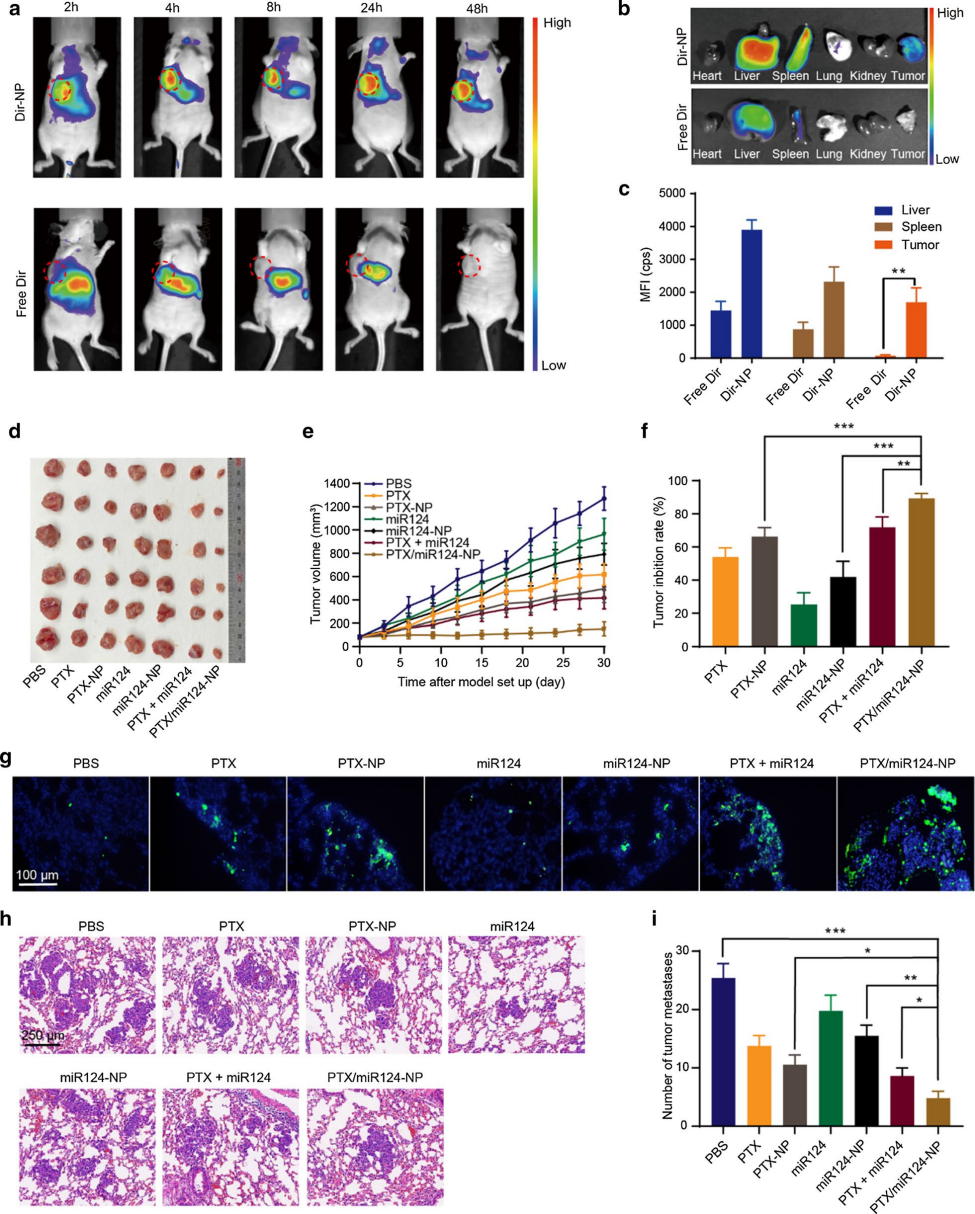
Therapeutic efficiency of PTX/miR124-NP in mice bearing MDA-MB-231 tumours. **a** Fluorescence images of mice at 2, 4, 8, 24 and 48 h after the intravenous injection of free Dir and Dir-NP. **b** Ex vivo fluorescence images of tumours and major organs 48 h after administration. **c** Quantified fluorescence intensity of the tumour and major organs (MFI, mean fluorescence intensity). **d** Images of tumours at the end of treatment (n = 6 for all groups). **e** The profiles of variations in the tumour volumes. **f** Tumour inhibition rate. **g** TUNEL analysis of tumour cell apoptosis. The green dot represents apoptotic cells. **h** Representative images of lung tissue sections from different treatment groups evaluated using H&E staining. **i** Quantitative analysis of the pulmonary metastatic nodules

Good targeting is essential for an excellent nanodelivery device. The in vivo biodistribution of CaP/LNS was investigated in an orthotopic tumour mouse model. By monitoring the fluorescent signal of Dir in mice, we found that free Dir was mainly enriched in the abdomen, and the fluorescent signal gradually weakened after 24 h. In contrast, the fluorescent signal remained strong in mice injected with Dir-NP after 48 h, especially at the tumour site (Fig. 5a, b). In addition, ex vivo fluorescence images of the tumour and major organs showed similar results (Fig. 5c), which demonstrated the good tumour targeting ability of CaP/LNS.

Next, we evaluated the therapeutic efficacy of PTX/miR124-NP in an MDA-MB-231 orthotopic tumour mouse model by monitoring tumour growth. As shown in Fig. 5d-f, the antitumour effect of the miR124 solution was poor, but the effect was significantly improved when miR124 was encapsulated into CaP/LNS NPs. Similarly, the efficacy of the PTX-NP group was higher than free PTX. Furthermore, the antitumour effects on the groups treated with the combination of PTX and miR124 were significantly stronger than on groups treated with the single agent, and PTX/miR124-NP exerted the strongest antitumour effect, with a tumour inhibition rate of 85.3%. Consistent with the change in tumour volume, TUNEL stained slices showed the highest proportion of apoptotic cells in the PTX/miR124-NP group (Fig. 5g).

The lung is the most common metastatic organ associated with TNBC, and lung metastasis is one of the leading causes of death [48]. Therefore, we evaluated the antimetastatic efficacy of PTX/miR124-NP using a lung metastasis mouse model. Severe lung metastasis was observed in the PBS group. Compared with the PBS group, the number of lung nodules in each treatment group decreased to a certain extent. The number of metastases in the NPs groups was significantly lower than those in the corresponding solution groups, and the lowest number was observed in the PTX/miR124-NP group (Fig. 5h, i). Overall, PTX/miR124-NP not only inhibited primary tumour growth, but also inhibited tumour metastasis.

### In vivo* evaluation of NPs safety*

**Fig. 6:**
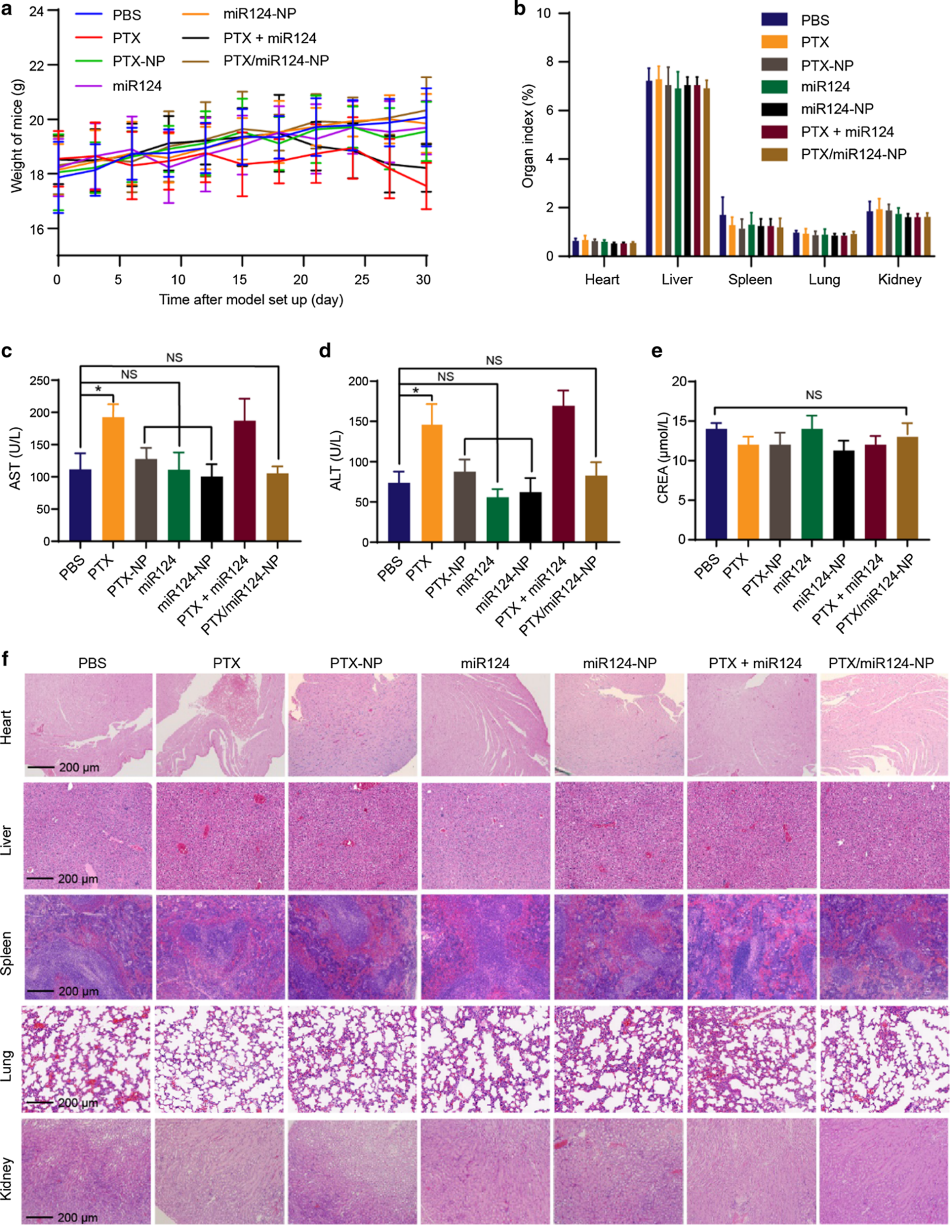
The safety of the PTX/miR124-NP treatment. **a** The profiles of variations in mouse body weight during treatment. **b** Organ indexes of the heart, liver, spleen, lung and kidney from mice following treatment with various formulations. **c, d** Impact on the serum AST (**c**) and ALT (**d**) levels. **e** Impact on the serum CREA levels. **f** Representative H&E staining images of the heart, liver, spleen, lung and kidney tissue sections collected from mice at the end of treatment. NS indicates no statistical significance, *P < 0.05

Good safety is essential for nanomedicine. The safety of the NPs was evaluated in the orthotopic tumour mouse model. The body weights of the mice in the PTX group and PTX + miR124 group showed a decreasing trend in the later stage of the experiment, which might be due to the systematic toxicity of PTX. The body weights of the mice in the PTX/miR124-NP group showed a slow increasing trend, suggesting that PTX/miR124-NP had no severe systematic toxicity (Fig. 6a). Compared with the PBS group, the organ indexes (heart, liver, spleen, lung and kidney) of the PTX/miR124-NP group showed no significant differences, which indicated that the NPs had no obvious organ toxicity (Fig. 6b). In addition, the serum levels of aspartate aminotransferase (AST) and alanine aminotransferase (ALT) were elevated in the free PTX and PTX + miR124 solution groups, while these parameters showed no abnormalities in the other treatment groups compared with the PBS control group (Fig. 6c, d). Based on these results, free PTX was toxic to the liver, and PTX loaded with CaP/LNS NPs not only improved the antitumour effect but also reduced the systemic toxicity. Regarding renal function, serum levels of creatinine (CREA) showed no significant differences between all groups (Fig. 6e). Moreover, as shown in Fig. 6f, the H&E staining did not reveal obvious pathological changes in the heart, liver, spleen, lung or kidney. These findings reflected the excellent safety of PTX/miR124-NP, suggesting that this formulation might be a potential therapeutic strategy for TNBC.

## Discussion

Traditional chemotherapy is gradually being withdrawn from clinical practice due to its unsatisfactory efficacy and severe side effects [3]. Paclitaxel is one of the most effective chemotherapy drugs for TNBC, but it suffers from a low effective rate of less than 30% [49]. Currently, numerous tumour driver genes have been identified through genome sequencing, and the synergism of chemotherapy and gene therapy has attracted increasing attention [21, 50]. The expression of miR124 is downregulated in various tumours. In triple negative breast cancer, miR124 not only inhibits tumour proliferation and metastasis but also sensitizes cancer cells to paclitaxel [12, 51]. Nanoparticles that are simultaneously loaded with different drugs/genes and precisely deliver cargos to tumour sites have become a promising vector for combined anticancer therapy [52]. Herein, we successfully developed a calcium phosphate composed lipid nanosystem (CaP/LNS) stepwise cleavable by specific microenvironment stimulus to codeliver PTX and miR124 for the treatment of TNBC.

NPs in the circulation are easily captured and cleaned by the reticular endothelial system (RES) alone [53]. In the nanosystem described in the present study, the surface of the NPs was modified with PEG to improve the stability of NPs and achieve long circulation and EPR effects. In order to overcome the obstacles of cell internalization and lysosome escape caused by PEGylation, we introduced a cleavable PEGylated modification through hydrazone bond that is responsive to the mildly acidic tumour microenvironment at the tumour site.

The EPR effect is still not sufficient to obtain a narrow target. An active targeting group for tumour cells is needed [54, 55]. HA is a water-soluble macromolecule, and endogenous HA participates in various biological functions in tumour cells [56]. Its receptor, CD44, is overexpressed in various tumour cells, including human triple negative breast cancer MDA-MB-231 cells, and is involved in tumour invasion and metastasis [57]. o-HA was introduced in the middle layer of the NPs as the active targeting group to further enhance the targeting ability of our system. When surface mPEG linked with hydrazone bonds was degraded by the acidic TME, the exposed o-HA block targeted the tumour cells via its affinity for CD44. After the NPs were internalized by the tumour cells, the disulfide bond was degraded by GSH, and the o-HA block was detached from the NPs, which enabled the o-HA fragments to inhibit the invasion and migration of tumour cells. Then, the NPs collapsed and decomposed, releasing PTX and miR124 to exert further antitumour effects. In the present study, the data showed that CaP/LNS had optimal environmental sensitivity and biocompatibility and efficiently delivered PTX and the miR124 to MDA-MB-231 cells. More importantly, CaP/LNS not only possessed a superior long term circulation effect but also exhibited excellent tumour targeting ability in vivo. Based on these results, we propose that CaP/LNS is an ideal anticancer drug/gene delivery system.

The EMT is a biological process in which epithelial cells are transformed into cells with mesenchymal phenotype [40]. In this process, epithelial cells gradually lose their epithelial differentiation characteristics, such as cell polarity and epithelial cell-to-cell junctions, and acquire migration, invasion and anti-apoptosis properties [58, 59]. Targeting the EMT is a promising strategy for cancer treatment. Both PTX and miR124 have been shown to reverse the EMT process [60–63]. In vitro experimental data showed that PTX/miR124-NP significantly induced apoptosis and inhibited the migration and invasion of MDA-MB-231 cells, and the antitumour effect of PTX/miR124-NP was stronger than NPs carrying a single agent. Similarly, codelivery of PTX and miR124 by CaP/LNS enhanced the reversal of the EMT process. Because miR124 improves the sensitivity of TNBC cells to PTX [14, 15], we reasonably conclude that the combination of PTX and miR124 exerts synergistic antitumour effects, which are partially attributed to the increased suppression of the EMT. Moreover, systemic administration of PTX/miR124-NP inhibited primary tumour growth and lung metastasis with excellent biosafety, and the efficacy of PTX/miR124-NP was obviously better than PTX-NP and mIR124-NP. Numerous studies have indicated that miR124 inhibits distant metastasis of TNBC [10, 13]. Therefore, we postulate that the decrease in lung metastatic nodules is caused by the cytotoxicity of PTX and the antimetastatic effect of miR124.

Altogether, PTX/miR124-NP displayed dual sensitivity to pH/GSH, dual tumour targeting properties, and excellent antitumour efficacy and safety, providing a potential approach for TNBC treatment. Unlike similar studies, PTX/miR124-NP achieved the synergy of the antitumour effects of PTX, miR124 and NPs components (o-HA), and the characteristic stepwise degradation increases its circulation time while avoiding the "PEG dilemma", thereby maximizing its antitumour efficacy. In addition, miR124 also reverses the PTX resistance of TNBC [14]. Therefore, PTX/miR124-NP may also have therapeutic potential for PTX-resistant TNBC. More importantly, other genes (such as miRNAs, siRNAs, etc.) can also be introduced into this system to achieve gene therapy or gene combination therapy for multiple tumours, which requires further research in the future.

## Conclusions

PTX/miR124-NP not only inhibited primary tumour growth but also inhibit tumour metastasis in mice bearing MDA-MB-231 tumour, without obvious toxicity to normal tissues. The inhibitory effects of PTX/miR124-NP were stronger than PTX and miR124 solutions and their single agent loaded CaP/LNS NPs. The EMT programme was significantly suppressed after PTX/miR124-NP administration. A synergistic antitumour effect was manifested between PTX, miR124 and the mPEG-HA-PC material. Collectively, the codelivery of PTX and miR124 by the CaP/LNS system might provide new ideas for the treatment of TNBC.

## Experimental section

### Materials

PEG-CHO and phosphatidyl choline (PC) were obtained from Ponsure Biotechnology Company (Shanghai, China). Adipic acid dihydrazide (ADH), cholesterol, hyaluronic acid (HA), 3,3′-thiodipropionic acid (TDPA), 4-dimethylaminopyridine (DMAP), N- (3-dimethylaminopropyl)-N′-ethylcarbodiimide hydrochloride (EDC), N,N'-dicyclohexyl carbon diimine (DCC) and thiazolyl blue tetrazolium bromide (MTT) were purchased from Sigma-Aldrich (St. Louis, USA). miR124, miRNA negative control and fluorescently labelled miRNA negative control were purchased from Ruibo Biotechnology Inc. (Guangzhou, China). Annexin V-FITC/PI and TUNEL apoptosis assay kits were purchased from Beyotime Biotechnology Inc. (Shanghai, China). PTX was obtained from Knowshine Co., Ltd. (Shanghai, China). The human triple negative breast cancer cell line MDA-MB-231 was obtained from the Cell Bank of the Chinese Academy of Sciences (Shanghai, China). Female BALB/c nude mice were obtained from Shanghai Slack Laboratory Animals Co., Ltd. (Shanghai, China) and were maintained under specific pathogen-free conditions.

### Conjugation of mPEG-HA

mPEG-CHO (1.5 g, 0.3 mmol, Mw = 5000) was dissolved in 45 ml of purified water. ADH (104.4 mg, 0.45 mmol) was dissolved in 15 ml of purified water and mixed with the mPEG-CHO solution. The mixture was stirred for 24 h and then dialyzed (MwCO = 3500) with water to remove small molecules. o-HA and EDC were added to the mPEG-ADH solution at different ratios. The pH was adjusted to 6–7. Oxygen was removed from the system with a nitrogen purge for 30 min. The mixture was sealed, stirred overnight and dialyzed (MwCO = 3500) with water to remove small molecules. The remaining solution was lyophilized and stored at -20 °C.

### Conjugation of mPEG-HA-PC

The esterification reaction was carried out by attaching 3,3′-thiodipropionic acid (TDPA) to the hydroxyl group of lecithin and attaching the carboxyl group at the other end to the hydroxyl group of HA via an esterification reaction to form mPEG-hydrazone bond-HA-disulfide bond-PC.

PC (0.375 g, 0.5 mmol) was dissolved in approximately 25 ml of DMSO, and 180 mg (1 mmol) of TDPA and 244 mg (2 mmol) of DMAP were added. The mixture was stirred under nitrogen for 30 min. A DMSO solution containing 420 mg (2 mmol) of DCC was added dropwise with stirring. The mixture was sealed and stirred overnight, followed by centrifugation. The supernatant was dialyzed (MwCO = 1000) alternately with water and DMSO and then centrifuged. The final supernatant was lyophilized to obtain PC-COOH. PC-COOH and mPEG-HA were dissolved in 25 ml of DMSO, followed by the addition of DMAP and DCC. The pH was adjusted to a weakly alkaline value. The mixture was stirred under nitrogen for 30 min to remove oxygen and water. The solution was then sealed, stirred overnight and centrifuged. The supernatant was dialyzed (MwCO = 3500) with DMSO to remove small molecules and then with ethanol–water to remove DMSO. The product was lyophilized to obtain mPEG-HA-PC.

### Optimization of the structure of mPEG-HA-PC

The ratio of mPEG to o-HA was selected by measuring the stability of the particle size and zeta potential, the release properties, the ability to inhibit the invasion of breast cancer cells, the compatibility with normal cells, and the acute toxicity reaction of PTX/miR124-NP.

### Preparation of PTX/miR124-NP

Ten milligrams of cholesterol and 50 mg of PC were added to the PTX dichloromethane solution as the oil phase. Next, miR124 and CaCl_2_ solutions were added and emulsified to obtain a W/O emulsion. Then, 10 mg of mPEG-HA-PC and Na_3_PO_4_ solution were added to purified water as the external aqueous phase and added to the W/O emulsion. After emulsification, the organic solvent was distilled under reduced pressure to obtain CaP complex lipid nanoparticles containing PTX and miR124 (PTX/miR124-NP).

### Stability of the particle size and zeta potential

The lyophilized nanoparticle (NP) powder prepared from the three materials was dispersed into water, buffers with pH values of 5.0, 6.5, and 7.4, and GSH solutions of 2 μM (pH 7.4), 2 mM, and 20 mM (pH 5.0) separately and adjusted to a volume of 5 ml. The NP solutions were sealed and shaken at 37 °C. The particle sizes and zeta potentials were measured at different times. The NPs at pH 7.4, pH 6.5 and in the presence of high GSH concentrations were centrifuged separately at 16,000 rpm for 1 h, and the precipitates were washed and lyophilized.

### Drug release properties

The miRNA negative control labelled with cy5 (cy5-miRNC) was used to determine the release of miR124 from the NPs. The cy5-miRNC-NP lyophilized powder was dispersed in 1 ml of DEPC water, sealed in a swollen dialysis tube (MWCO = 100,000) and immersed in 10 ml of medium. The release medium was the same as the medium used to assay the stability of the particle size and zeta potential. The release occurred at 37 °C and 100 r/min. At every preset time point, 2 ml of the external solution was removed, and an equal volume of fresh medium was added. Then, the same concentration of free cy5-miRNC was used as a control. Fluorescence intensity was measured using a fluorescence spectrophotometer (Hitachi F-2700, Japan), the concentration of cy5-miRNC was calculated, and a release profile was plotted.

The PTX-NP lyophilized powder was dispersed in 0.5 ml of DEPC water, sealed in a swollen dialysis tube (MWCO = 3500) and immersed in 19.5 ml of medium. The release medium was the same as the medium used to determine the stability of the particle size and zeta potential and contained sodium salicylate (1 mol/L). Release occurred at 37 °C and 100 r/min. At every preset time point, 0.2 ml of the external solution was removed, and an equal volume of fresh medium was added. At the same time, the PTX solution with the same concentration was used as a control. The PTX concentration was determined using HPLC, and the release profile was plotted.

### Scratch test

MDA-MB-231 cells were seeded in 6-well plates and incubated with serum-free medium overnight. The plates were scratched, the floating cells were removed, and the adherent cells were then cultured with the corresponding substances. The o-HA concentrations were 0.5, 1.5, and 4.5 mg/ml. The material solution and the NP concentration were adjusted with the HA block (1.5 mg/ml). The miR124 concentration was 130 pmol/ml. Photographs were taken with a microscope (Olympus IX51, Japan) at 0, 24, and 48 h. The areas of the scratches were measured using ImageJ software (version 1.8.0, NIH, USA). Reductions in the areas were measured to evaluate the inhibition of tumour migration.

### Cytocompatibility evaluation

Cytocompatibility was evaluated in LO2 normal human liver cells with an MTT experiment. The cells were plated in 96-well plates and incubated with NPs for 24, 48 and 72 h. The medium was exchanged for MTT (0.5 mg/ml) in DMEM and incubated for 3 h. The absorbance of MTT was determined with a microplate reader (Beckman, USA) at 490 nm.

### The synergistic antitumour effect of PTX and miR124

The MTT method was used to determine the cytotoxicity of PTX, miR124, and their mixed solution in MDA-MB-231 cells. The synergy factor (CDI) was calculated using the following formula to select the optimal ratio: CDI = AB/A·B, where A is the absorbance of the PTX-treated group, B is the absorbance of the miR124-treated group, and AB is the absorbance of the group treated with their mixture at the same concentrations.

### Preparation and characterization of the optimized PTX/miR124-NP

Through screening, mPEG-HA-PC 2:1 was chosen for subsequent studies. Then, we optimized the method for preparing the NPs by performing orthogonal experiments and single-factor experiments. The NPs were prepared as described below. The oil phase contained 50 mg of PC, 10 mg of cholesterol and 3.15 mg of PTX dissolved in 0.5 ml of dichloromethane; miR124 was dissolved at a concentration of 10 nmol/50 μl in DEPC water. The internal water phase was mixed with 65 μl of the miR124 solution, 50 μl of the CaCl_2_ solution (200 mM) and 85 μl of DEPC water for 5 min. Then, the top oil phase was added to the internal water phase. The mixture was emulsified in an ice bath (2 s-2 s-1 min, 65 W) to obtain a primary emulsion. The external aqueous phase was prepared with 10 mg of mPEG-HA-PC, 75 μl of 3 mM Na_3_PO_4_ and 5 ml of DEPC water. The external aqueous phase and primary emulsion were mixed and emulsified in an ice bath (2 s-2 s-2 min, 130 W). The organic solvent was removed under reduced pressure. Finally, the NPs were adjusted to a volume of 5 ml with water.

The particle size and zeta potential were measured with a laser scattering particle size analyser (Malvern, USA). The morphology of the NPs was observed using transmission electron microscopy (FEI Co., USA) after negative staining with phosphotungstic acid. Drug release was also investigated as described above. The encapsulation efficiencies (EEs) of PTX and the miRNA were measured after centrifugation (16,000 rpm/min for 1 h). EE = (amount of drug entrapped in NPs/total amount of drug) × 100%.

### *Cellular uptake of NPs *in vitro

FAM-miRNC was entrapped in CaP/LNS (FAM-miRNC-NP) as a fluorescent probe to observe the cellular uptake of NPs by MDA-MB-231 cells. Cells were incubated with PBS, FAM-miRNC or FAM-miRNC-NP for 5 h. Cells were washed with PBS three times and fixed with 4% formaldehyde for 15 min at room temperature. Subsequently, the nuclei were counterstained with DAPI (blue) for 15 min, and the internalization of the NPs by MDA-MB-231 cells was visualized using CLSM (Olympus, Japan). In addition to CLSM, the internalization of NPs by MDA-MB-231 cells was also analysed using flow cytometry (BD Biosciences, USA).

### Cell migration and invasion

A Transwell chamber (8 μm) was used to evaluate the migration and invasion of MDA-MB-231 cells. For the migration experiment, MDA-MB-231 cells were cultured in serum-free medium for 12 h before trypsin digestion. The cells were suspended in serum-free medium and adjusted to a density of 8 × 10^5^ cells/ml. Each well contained 0.5 ml of 15% serum. Then, 100 μl of the cell suspension were added to the chamber and placed in the well. Next, 0.1 ml of drug solution in serum-free medium was added to the cell suspension and cultured for 48 h. The cells that passed through the chamber membrane were determined using the MTT method. For the invasion experiment, the Transwell chambers were precoated with a layer of Matrigel. The other steps were the same as those in the migration experiment.

### Apoptosis analysis

Annexin V–FITC/PI double staining assays were performed to evaluate cell apoptosis. MDA-MB-231 cells were plated in 6-well plates overnight and then treated with different formulations (PBS, PTX solution, miR124 solution, PTX-NP, miR124-NP, PTX and miR124 solution mixture or PTX/miR124-NP) for 48 h. Subsequently, cells were stained with Annexin V-FITC/PI according to the manufacturer’s instructions for the flow cytometry analysis.

### Western blotting

Cells were treated with different drugs as described above. Then, the cells were washed with PBS twice to remove exogenous proteins, and total protein was extracted. Western blotting was performed using standard procedures. Briefly, total proteins (20 μg per well) were separated on SDS–PAGE gels and transferred to PVDF membranes by electroblotting. These membranes were incubated with primary antibodies against MMP-9 (Abcam, ab38898), E-cadherin (CST, 5296S), N-cadherin (CST, 13116S), Vimentin (CST, 5741S) and GAPDH (Abcam, ab59164) at 4 °C overnight and then incubated with HRP-conjugated anti-mouse/rabbit secondary antibodies (Abcam, ab205719; CST, 7074S) for 1 h at room temperature. The protein bands were visualized using a Bio-Rad Imaging System (Bio-Rad, USA). The expression levels of the proteins were quantified using Image Lab software (version 3.0, Bio-Rad, USA).

### Biodistribution of NPs

MDA-MB-231 cells (1 × 10^6^) were inoculated into the fourth fat pad to establish the orthotopic tumour mouse model of TNBC. Free Dir and Dir-NP (0.5 mg/kg Dir) were injected via the tail vein (3 mice per group). Fluorescence images were acquired at different time points using a fluorescence imaging apparatus (Berthold Technologies, Germany). At 48 h post injection, the tumours and major organs were harvested to further analyse the fluorescence intensity of the different tissues ex vivo.

### *The antitumour effects and safety of PTX/miR124-NP *in vivo

The orthotopic tumour mouse model was used to evaluate the effect of PTX/miR124-NP on inhibiting primary tumour growth. When the tumour volume reached 75–100 mm^3^, these mice were randomly divided into 7 groups (PBS, PTX solution, miR124 solution, PTX-NP, miR124-NP, PTX and miR124 solution mixture and PTX/miR124-NP) and administered the respective treatments (PTX dose of 1 mg/kg, miR124 dose of 200 nmol/kg) via the tail vein once every 3 days for 4 weeks. The volume of the tumours and the weight of the mice were measured before each administration. On the third day after the last administration, blood was collected to measure ALT and AST levels using the corresponding biochemical assay kits according to the manufacturer’s instructions. The tumours were subjected to a TUNEL assay to assess apoptosis. Major organs (heart, liver, spleen, lung and kidney) were removed for H&E staining.

### *The antimetastatic effect of PTX/miR124-NP *in vivo

The lung metastasis model was established by injecting the MDA-MB-231 cell suspension (1 × 10^6^) into nude mice via the tail vein. Five days after the intravenous injection of MDA-MB-231 cells, the mice were administered the respective formulations (PTX dose of 1 mg/kg, miR124 dose of 200 nmol/kg) via the tail vein once every 3 days for 2 weeks. The lungs were sliced and stained with H&E to evaluate metastasis. The number of metastases was counted, and the tumour inhibition rate was calculated.

### Statistical analysis

The results are presented as the mean ± SD. The level of significance was determined by one-way ANOVA with Tukey’s pairwise comparison using SPSS software (version 17.0, IBM Inc., USA). P < 0.05 was considered statistically significant.

## Supplementary Information

**Additional file 1: Figure S1.** The synthetic route of mPEG-HA-PC. **Figure S2.** ^1^H NMR spectra of mPEG-HA. **Figure S3.** FTIR spectra of mPEG-HA. **Figure S4.** FTIR spectra of mPEG-HA-PC in different ratios. **Figure S5.** The NPs inhibit the migration of MDA-MB-231 cells. **Figure S6.** Synergistic cytotoxicity of PTX and miR124 on MDA-MB-231 cells.

## Data Availability

All data and materials are included in this published article and its additional files.
