# Supplementary material for: A paclitaxel and microRNA-124 coloaded stepped cleavable nanosystem against triple negative breast cancer
Source: J Nanobiotechnology. 2021 Feb 25;19:55. doi: 10.1186/s12951-021-00800-z (PMC7905927; doi:10.1186/s12951-021-00800-z)
Supplement: Supplementary file 1 — Additional file 1: Figure S1. The synthetic route of mPEG-HA-PC. Figure S2. 1H NMR spectra of mPEG-HA. Figure S3. FTIR spectra of mPEG-HA. Figure S4. FTIR spectra of mPEG-HA-PC in different ratios. Figure S5. The NPs inhibit the migration of MDA-MB-231 cells. Figure S6. Synergistic cytotoxicity of PTX and miR124 on MDA-MB-231 cells. [file 12951_2021_800_MOESM1_ESM.docx]

**Additional information**

**A paclitaxel and microRNA-124 coloaded stepped cleavable nanosystem against triple negative breast cancer**

Chuanrong Chen^1†^, Ming Shen^1,2†*^, Hongze Liao^3*^, Qianqian Guo^4^, Hao Fu^4^, Jian Yu^1*^, Yourong Duan^1*^

^1^State Key Laboratory of Oncogenes and Related Genes, Shanghai Cancer Institute, Renji Hospital, School of Medicine, Shanghai Jiao Tong University, Shanghai 200032, China

^2^NHC Key Laboratory of Reproduction Regulation (Shanghai Institute of Planned Parenthood Research), Fudan University, and Shanghai Engineer and Technology Research Center of Reproductive Health Drug and Devices, Shanghai, China

^3^Research Center for Marine Drugs, State Key Laboratory of Oncogene and Related Genes, Department of Pharmacy, Ren Ji Hospital, School of Medicine, Shanghai Jiao Tong University, Shanghai, China

^4^State Key Laboratory of Oncogenes and Related Genes, Renji Hospital, School of Biomedical Engineering, Shanghai Jiao Tong University, Shanghai, China





**Fig. S1** The synthetic route of mPEG-HA-PC. The mPEG and o-HA were linked *via* a hydrazone bond to construct mPEG-HA, and then lecithin was attached to mPEG-HA by a disulfide bond to form mPEG- [hydrazone](javascript:;) [bond](javascript:;) -HA -disulfide bond-PC.


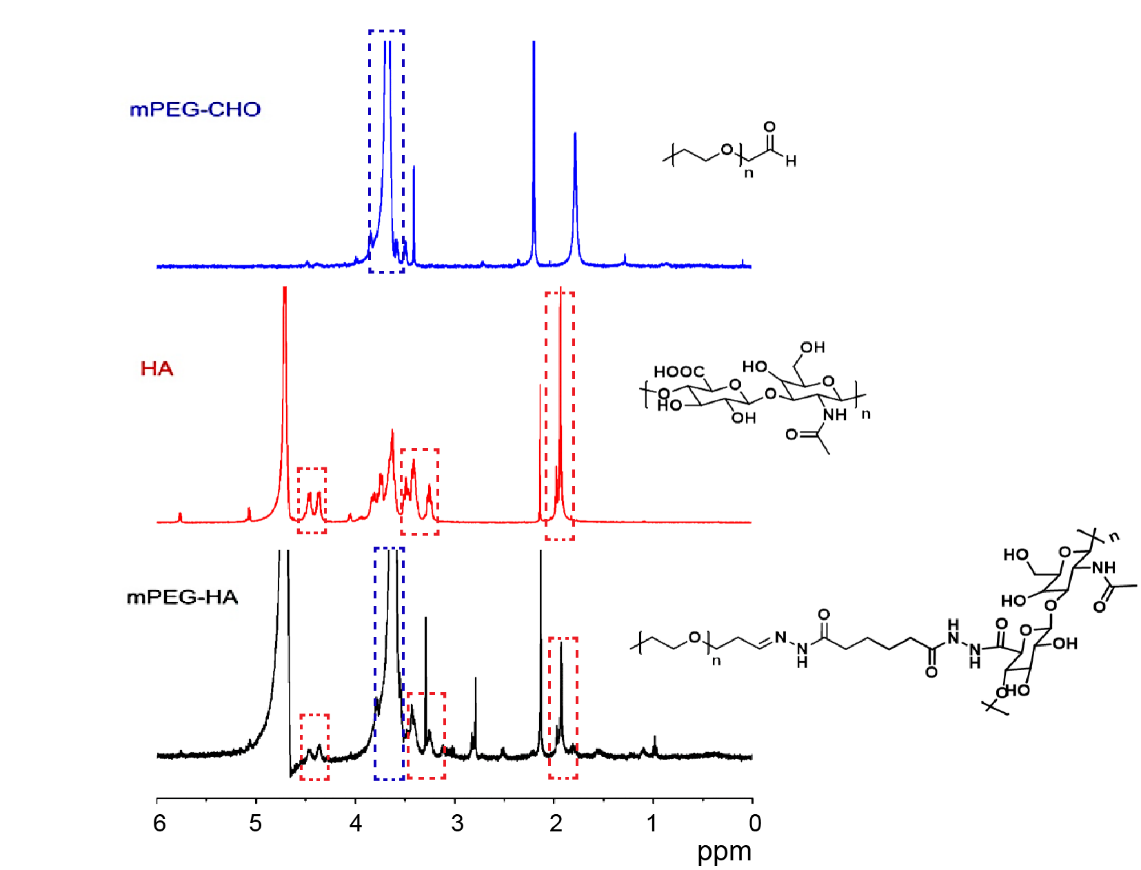


**Fig. S2** ^1^H NMR spectra of mPEG-HA.


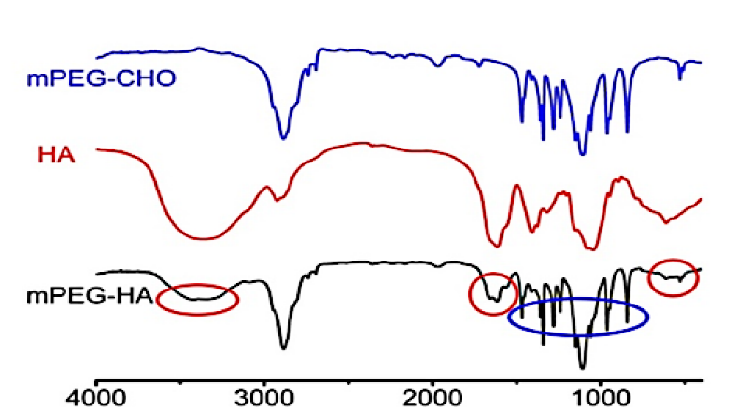


**Fig. S3** FTIR spectra of mPEG-HA.


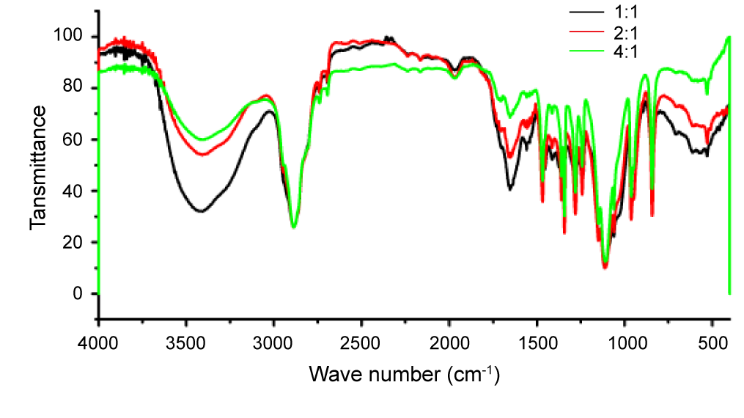


**Fig. S4** FTIR spectra of mPEG-HA-PC in different ratios. The FTIR absorption peaks at 1720 cm^-1^ corresponded to the C=O of ester.


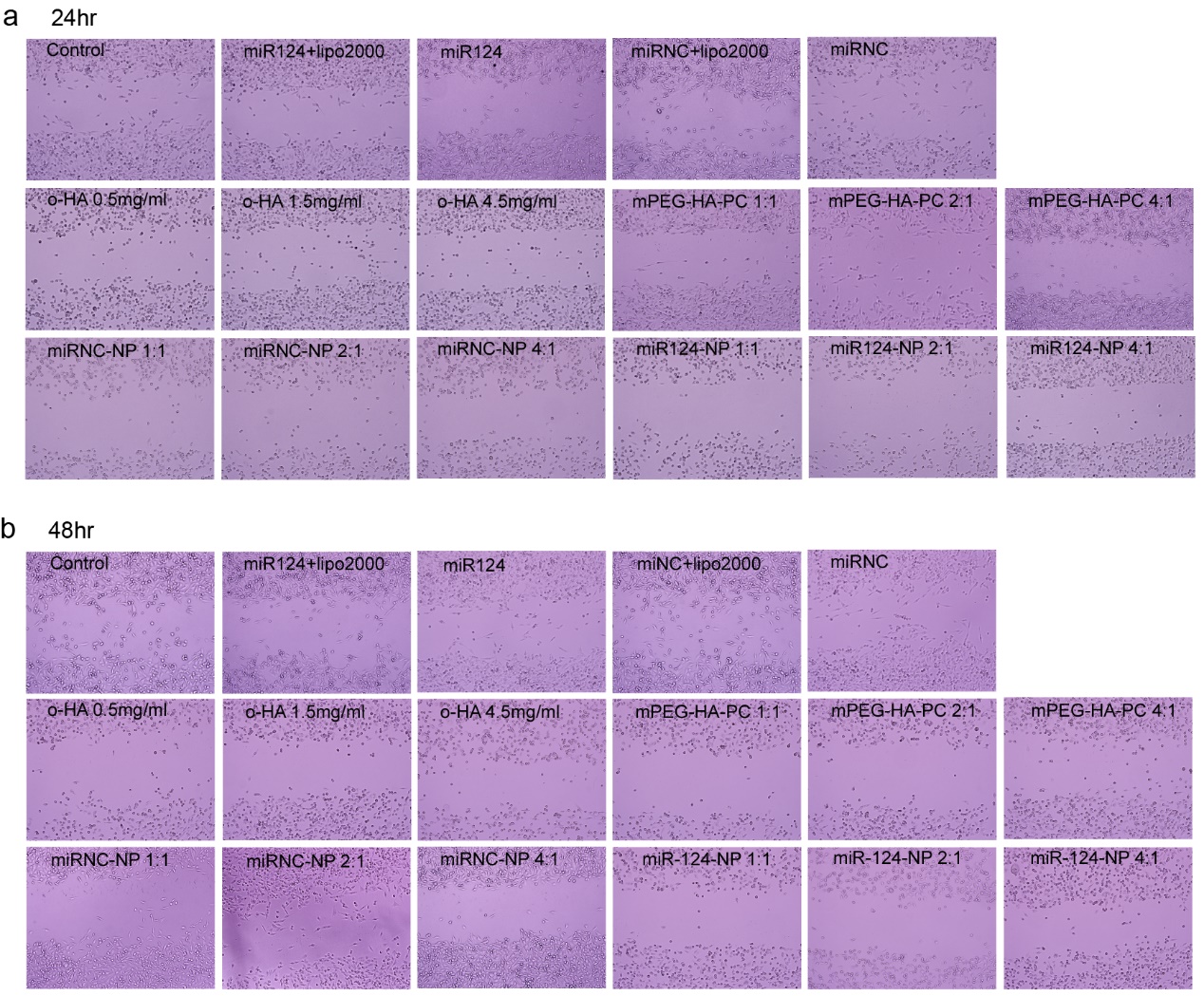


**Fig. S5** The NPs inhibit the invasion of MDA-MB-231 cells. **a, b** Representative wound-healing pictures of MDA-MB-231 cells treated with miR124 + Lipo2000, miR124, miRNC + Lipo2000, miRNC, o-HA (0.5 mg/ml, 1.5 mg/ml, 4.5 mg/ml), mPEG-HA-PC (1:1, 2:1, 4:1), miRNC-NP (1:1, 2:1, 4:1) and miR124-NP (1:1, 2:1, 4:1) for 24 h and 48 h.


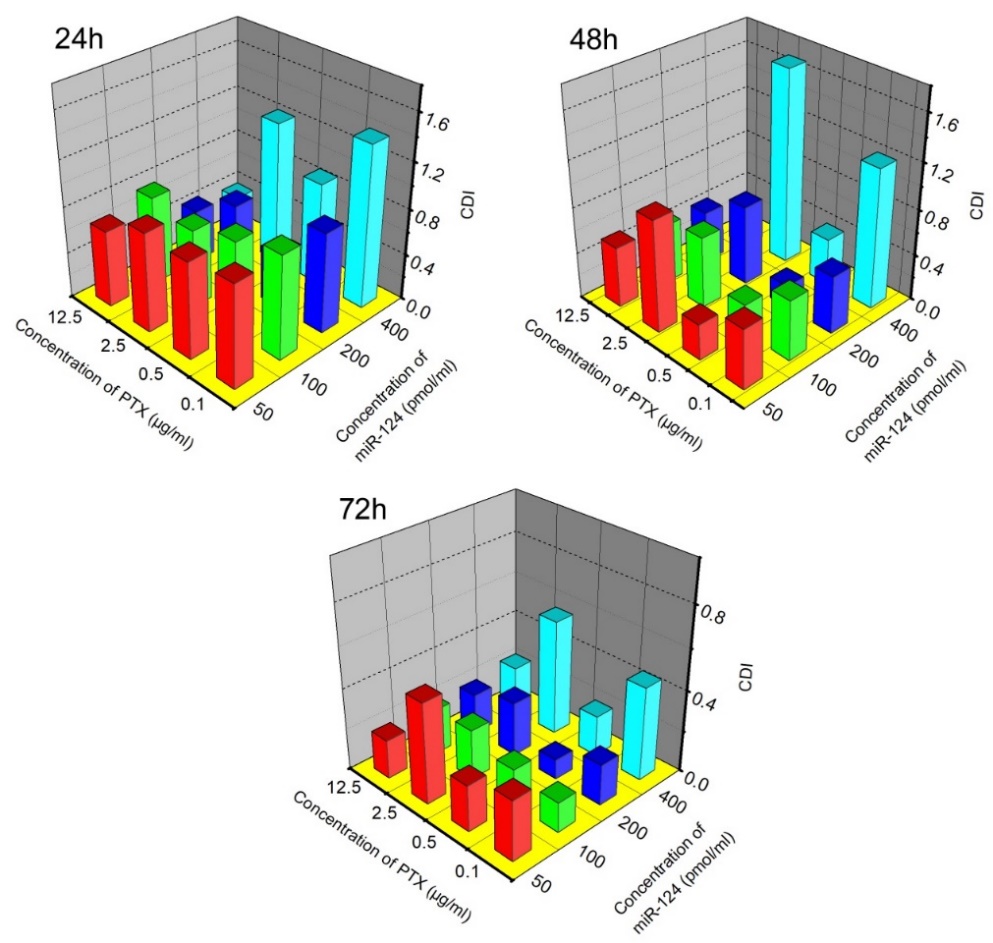


**Fig. S6** Synergistic cytotoxicity of PTX and miR124 on MDA-MB-231 cells.
